# The Role of Pectin Hydrogel Systems Plus Iodine and Phyto Extract in Second-Degree Burn in Rats

**DOI:** 10.5152/eurasianjmed.2024.24550

**Published:** 2024-10-01

**Authors:** Kubra Aliyeva, Abdulmecit Albayrak, Erdem Toktay, Emir Enis Yurdgulu, Yasin Bayir

**Affiliations:** 1 Department of Pharmaceutical Technology and Management, Azerbaijan Medical University Faculty of Pharmacy Baku, Azerbaijan; 2 Department of Pharmacology, Ataturk University Faculty of Medicine, Erzurum, Türkiye; 3 Department of Histology and Embryology, Kafkas University Faculty of Medicine, Kars, Türkiye; 4 Department of Biochemistry, Ataturk University Faculty of Pharmacy, Erzurum, Türkiye

**Keywords:** Burn, iodine, pectin-based hydrogel systems, plant extract, rat

## Abstract

**Background:**

Skin injuries, such as burns, can result in an open wound that can lead to the deterioration of the skin, which acts as a protective barrier against external agents, and can cause serious health problems. Pectin, a plant-derived polysaccharide, is a suitable candidate for wound care due to its gel-forming ability and biocompatibility. Pectin absorbs wound exudates to form a soft gel and is known for its strong anti-inflammatory effects. Pectin-based hydrogel systems promote wound healing by promoting fibroblast proliferation and keratinocyte migration.

**Methods:**

: In the study, 60 male Wistar albino rats were used, and 6 different treatment groups were formed: Healthy control, burn control, silver sulfadiazine, pectin-based hydrogel system, pectin-based hydrogel system + iodine, pectin-based hydrogel system + plant extract. Rats with burn wounds were treated for 21 days, and the wound healing process was evaluated by macroscopic, biochemical, and histopathological analysis.

**Results:**

The results showed that the pectin-based hydrogel system, iodine, and plant extracts promoted healing in burn wounds. Especially in the groups treated with silver sulfadiazine and plant extracts, the development of granulation tissue and regeneration of collagen fibers showed significant improvement. Furthermore, the pectin-based hydrogel system is thought to contribute to wound healing by maintaining skin moisturization. Vascular endothelial growth factor (VEGF) and transforming growth factor-β (TGF-β) levels decreased in the treatment groups compared to the burn group, and it was determined that these factors play an important role in wound healing.

**Conclusion:**

This study shows that pectin-based hydrogel systems can be an effective alternative in burn treatment, and their healing effects can be enhanced by the addition of iodine and plant extracts.

**Main Points**

- Vascular endothelial growth factor (VEGF) and transforming growth factor-β (TGF-β1) are important in the early stages of wound healing, and these levels were observed to decrease in parallel with healing in the treatment groups.
- Collagen accumulation plays an important role during burn healing, and type I collagen levels decreased in the treatment groups.
- In histopathological analyses, significant improvement was observed in the development of granulation tissue and re-formation of collagen fibers in the groups containing silver sulfadiazine (SLV) and plant extracts.
- Pectin-based hydrogel system (PHS) was thought to contribute to wound healing by maintaining skin moisturization.
- This study shows that the combination of pectin hydrogel systems with iodine and plant extracts may be a new and effective treatment method in the burn healing process.

## Introduction

The skin is the largest organ in our bodies and serves as a protective barrier against many environmental elements. However, skin injuries, such as burns, can cause a breakdown of this important protective barrier and result in an open wound that can lead to serious health problems. Burn injuries result from skin contact with a hot source.^1^ Burn injuries can be caused by high temperatures, electricity, friction, radiation, and chemicals.^2^ Burn injuries differ, and the increase in body surface area impacted by a burn injury influences wound morbidity and patient mortality.^3^ Other critical parameters that directly impact the severity of the damage are the location of the burn, temperature, and duration of exposure to the heat source, which additionally has a synergistic effect.^4^

Treatment of burn wounds includes goals such as restoring skin integrity, reducing the risk of infection, and accelerating the healing process. For this purpose, various treatment methods and products have been developed for wound care. In recent years, the potential of natural components in burn treatment has attracted more and more attention. Naturally derived substances such as herbal extracts and natural polysaccharides can support the healing process of burn wounds and offer an alternative or supportive approach to traditional treatment methods.^5^ Many plants and plant-derived products have been shown to have strong wound healing activity.^6^

Wound dressings today are made of polymeric materials such as alginates, films, foams, and hydrocolloids. However, there are restrictions to using these materials as wound dressings. For example, certain films are non-absorbent, enabling wound exudates to collect; foams generate an opaque coating that complicates wound monitoring and hydrocolloids have substantial exudate leakage.^7^

Pectin is a polysaccharide of plant origin and a suitable candidate for wound care due to its biocompatibility and gel-forming ability. Plant cell walls include pectin, which is mostly composed of galacturonic acids and carboxyl moieties that are partially esterified with methoxy groups. It may be formed into hydrogels to retain and transport proteins, medicines, and cells.^8^ Pectin’s amorphous nature makes it appropriate for usage in skin tissue applications, while its hydrophilic component combines with wound exudates to form a soft gel responsible for wound exudate removal and management.^9^ The high hydrophilicity of pectin, which has strong anti-inflammatory effects, is similar to that of other wound-healing polyanions. This makes it preferable in the treatment of chronic diabetic wounds and burns.^10,11^

Hydrogel wound dressings can absorb wound exudates by promoting keratinocyte migration and fibroblast proliferation.^7,12^ Its structure can prevent infections in wounds; it also facilitates the transport of drugs and bioactive molecules such as antibiotics to the wound center. With its water content similar to normal healthy tissue, it provides the flexibility required to adapt to wounds in different parts of the body. It also acts as a wound dressing that does not interfere with the tissue repair process and does not need to be replaced regularly.^13^ Therefore, this study aims to investigate the ability of a pectin-based hydrogel system to promote wound healing in rats with a second-degree burn model.

## Material and Methods

### Animals and Groups

In this study, 60 male Wistar albino rats were used. All stages of our study were approved by the Atatürk University Animal Experiments Ethics Committee (AUHADYEK) dated 08.11.2022 and numbered E-75296309-050.01.04-2200366217 in accordance with ethical rules. Animals with an average weight of 200-250 g were randomly divided into 6 groups. These 6 groups consisted of healthy, burn control, silver sulfadiazine (SLV) as a positive control, Pectin-based hydrogel system (PHS) + iodine, and PHS + phyto extract (Table 1).

### Preperation of Pectin-based Hydrogel System and Phyto extracts

Pectin-based hydrogel system (PHS) prepared with pectin-gelatin complexes was used as a carrier. Liquid extracts obtained from iodine and wound-healing plant complexes added to PHS were used.

Pectin-based hydrogel system (PHS) was prepared with a new method, and a patent was obtained for the technology used.^14^ At the same time, a new technology was used in the preparation of pectin used in this study, and a patent was obtained for this technology.^15^ Pectin-based hydrogel system (PHS) was prepared by adding pectin gelatin complex, boric acid, Tween-80, glycerin, and propylene glycol in a ratio of 1 : 10. Chlorhexidine solution of 0.01% was preferred as an astringent.

The therapeutic PHS + iodine complex was prepared by adding a 0.6% iodine-containing solution with antiseptic, blood-thinning, and wound healing effects to PHS.

In the preparation of the second wound-healing PHS + phyto extract group, liquid extracts obtained from wound-healing plant complexes were used as active ingredients. Folium Plantaginis - DBX (QOST 2578-67, AR-20 g), Flores Calendulae - DBX (QOST 6077-80, AR-20 g), Herba Hyperici - DBX (QOST 15161-93, AR-20 g) in the phytocomplex containing biologically active compounds, Folium Urticae - DBX (QOST 12529-67, AR-10 g), Radix Glycyrrhizae - DBX (QOST 22839-88, AR-10 g) were purchased from Herba Flora company. Folium Juglans (20 g) was collected from Kuba, Azerbaijan in May-June. Liquid extracts were obtained from the wound-healing plant complexes by using 70% ethanol. The 70% extract mixture and false needle oil were combined with PHS, and PHS + phyto extract complex was prepared.

### Experimental Protocol for Wound Healing in Rats

At the beginning of the experiment, animals were first anesthetized. Ketamine and xylazine (80 : 20) were used for anesthesia. Then, the hairs on the back and flanks of the animals were shaved at zero level. A straight line was drawn on the back of the animal with the help of a ruler. A burn was created on the right side of the back of the animal based on the center of the drawn line. Burn wounds were created using the “comb burn” model described by Regas and Ehrlich.^16^ A brass comb (teeth: 2×1×2 cm, interdental spaces: 2×0.5×2 cm) was kept in a beaker of boiling water at 100°C for 10 minutes and then held on the animal for 15 seconds without applying any force to create a second-degree burn. This procedure was performed on the right side of each animal. Silver sulfadiazine, PHS, PHS + iodine, and PHS + phyto extract were applied topically to these wounds according to the group. This application was repeated every day for 21 days. Due to severe pain from the beginning of the experiment, novalgin (10 mg/kg) was added to the drinking water of the animals for 3 days as a painkiller. The day of the experiment was considered as day zero and the animals were anesthetized with ketamine and xylazine (80 : 20) anesthesia on days 3, 7, 14, and 21, and their photographs were taken. All animals were sacrificed at the end of the experiment. During the experiment and on the last day of the experiment, the ImageJ program was used to score the macroscopic healing analysis.^17^ Wound healing rates were calculated using the photographs of the animals.

### Biochemical Analysis

Incisional biopsies of 5 mm in size were taken from the burn areas on the right side of the rats under anesthesia, on the 21st day, right next to the wound healing area, and were stored at −80°C until analysis. After the tissues were homogenized with liquid nitrogen, 30 mg of tissue was homogenized in a liquid medium with 1 mL of PBS buffer via Qiagen Tissulyser II.^18^ It was centrifuged in a refrigerated centrifuge at 2200 *g* for 5 minutes, and the supernatants were transferred to another tube. The supernatant was used as a sample for the measurement of the tumor necrosis factor-alpha (TNF-α), TGF-β, VEGF, and Collagen Type 1 (Col1). The analyses were carried out following the manufacturer’s instructions: FineTest Rat TNF-α (Cat.No: ER1393), FineTest Rat TGF-β (Cat.No: QT-ER1378), FineTest Rat VEGF (Cat.No: ER0069), and FineTest Rat Col1, (Cat.No:ER0851), respectively. Total protein levels were analyzed by the Lowry Method, using commercial protein standards (Sigma Aldrich, TP0300).^19^ The results were expressed as mean ± SD of concentration (pg/mg protein) for all groups.

### Histological Analyses

The tissue samples were fixed in a 10% solution of formalin for 48 hours. Following that, routine tissue examinations were carried out. The tissues were first rinsed in running water for 20 minutes to remove the formalin. The water was then removed from the tissues by passing them through a sequence of increasing alcohol concentrations (50, 60, 70, 80, 96, and 99) for an hour. The tissue samples were then rendered transparent by passing them through a three-step xylene series for 15 minutes each. Finally, the tissues were immersed in warm paraffin and baked in an oven at 60°C for two 1-hour changes before the paraffin was impregnated into the tissue. The tissue samples were then blocked and sectioned. A microtome was used to cut paraffin blocks to a thickness of 5 μm. The sections were stained using Masson’s Trichrome stain. The staining protocol is as follows. The paraffin was removed by keeping the sections in an oven at 60°C and the tissue was well fixed on the slide. Subsequently, the paraffin was completely removed by soaking it in a xylene solution for 45 minutes. The slides were then soaked in 100% alcohol, 95% alcohol, and 70% alcohol solutions for 2 minutes each and washed in distilled water for 5 minutes. Slides were refixed in Bouin’s solution at 56°C for 1 hour to improve the staining quality and washed again in running tap water for 10 minutes to remove the yellow color. Nucleus staining was then performed in iron haematoxylin working solution for 10 minutes and the excess stain was removed by washing in warm tap water for 10 minutes. This was followed by staining in Biebrich red acid fuchsin solution for 10 minutes and washing again in distilled water. Subsequently, the sections were kept in phosphomolybdic-phosphotungstic acid solution for 10-15 minutes or until the collagen was no longer red. The sections were then transferred directly (without rinsing) to aniline blue solution and left for 5 minutes. Rinse briefly in distilled water and soaked in 1% acetic acid solution for 2 minutes. After rinsing again in distilled water, 95% ethyl alcohol, absolute ethyl alcohol, and xylene were quickly passed and the slide surface was covered with a coverslip. After waiting for preservation, the slides were examined and photographed with an Olympus CX 21 camera attachment microscope. Finally, the images were composited with Photoshop CS5.

### Statistical Analysis

One-way analysis of variance (ANOVA) was used for the statistical analysis. As a post-hoc test, the Least Significant Differences (LSD) multiple range test was used for biochemical data, and the Tukey test was used for stereological data by utilizing the IBM-SPSS software package version 20.00 (IBM SPSS Corp.; Armonk, NY, USA). Significance was attributed to *P*values < .05. The reported results are expressed as mean values ± standard deviation (SD), and the data are derived from 10 rats in each group.

## Results

### Wound Healing Results

**Figure 1.**
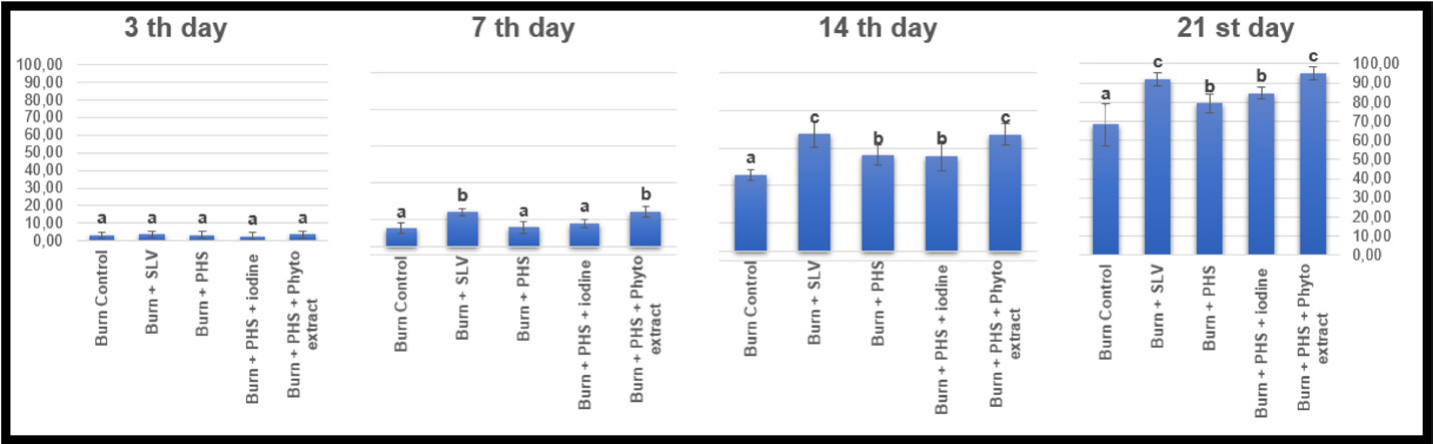
Recovery rates by days. PHS, pectin-based hydrogel system; SLV, silver sulfadiazine.

**Figure 2.**
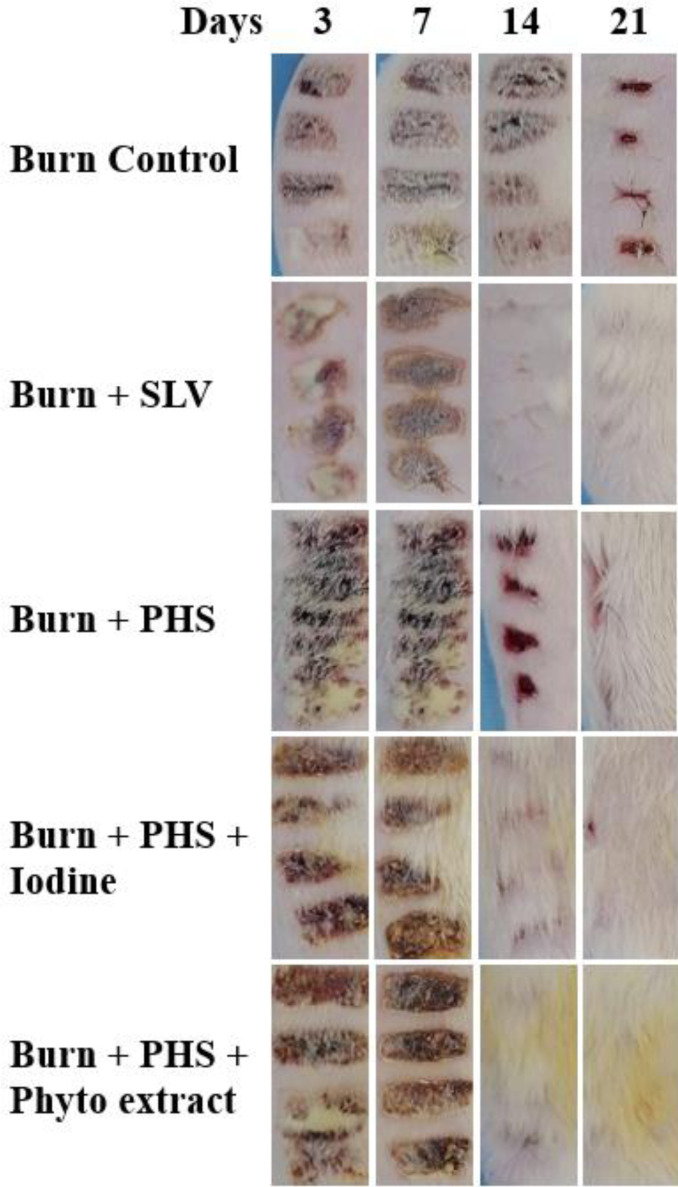
Macroscopic healing images by days. PHS, pectin-based hydrogel system; SLV, silver sulfadiazine.

Photographs taken on the 3rd, 7th, 14th, and 21st days of the burn, and the healing percentages are given in Figures 1 and 2 in comparison with the control groups. Compared to the control group, although very little macroscopic improvement was observed in the silverdin group compared to the other drugs on the 3rd day, statistically, none of the groups showed a significant improvement compared to each other. On the 7th day, when the wound healing was evaluated between the groups, it was determined that the SLV and phyto extract groups showed a statistically significant improvement compared to the control group. On the 14th and 21st days, when the wound healing was evaluated between the groups, it was determined that there was a statistically significant improvement in the PHS, iodine, SLV, and phyto extract groups compared to the control group, but there was greater improvement in the SLV and phyto extract groups. Recovery rates and macroscopic photographs taken on the determined days were parallel (Figures 1-2). Since macroscopic observations, healing rates, and photographs showed protective results in terms of healing, biochemical healing parameter analyses and microscopic analyses were performed.

### Biochemical Analyses

#### Tumor Necrosis Factor-Alpha Results

**Figure 3.**
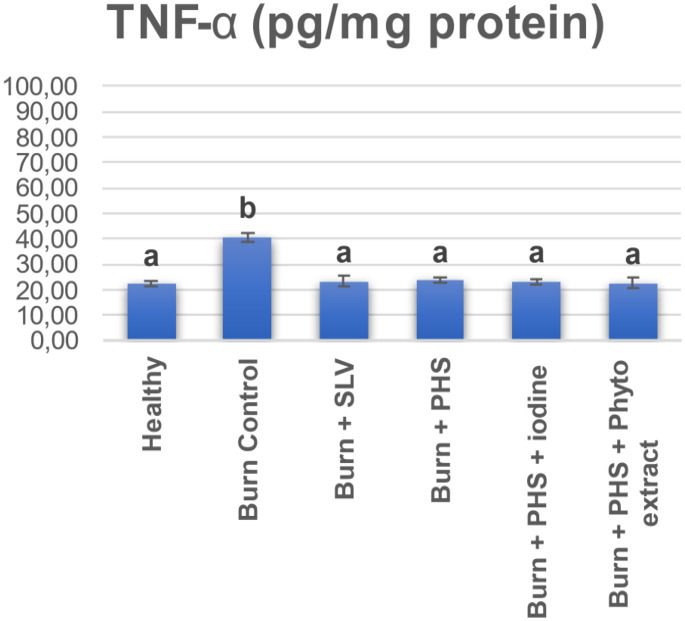
Tumor necrosis factor-alpha (TNF-α) results on skin tissue on day 21. PHS, pectin-based hydrogel system; SLV, silver sulfadiazine.

It was observed that the amount of TNF-α increased in the burn control group compared to the healthy group. This shows that the burn model is formed. When PHS and iodine groups were compared with the burn control group, TNF-α levels were found to be significantly decreased; in SLV and phyto extract groups, TNF-α levels were found to be very close to the healthy group (Figure 3).

#### Transforming Growth Factor-β Results

**Figure 4.**
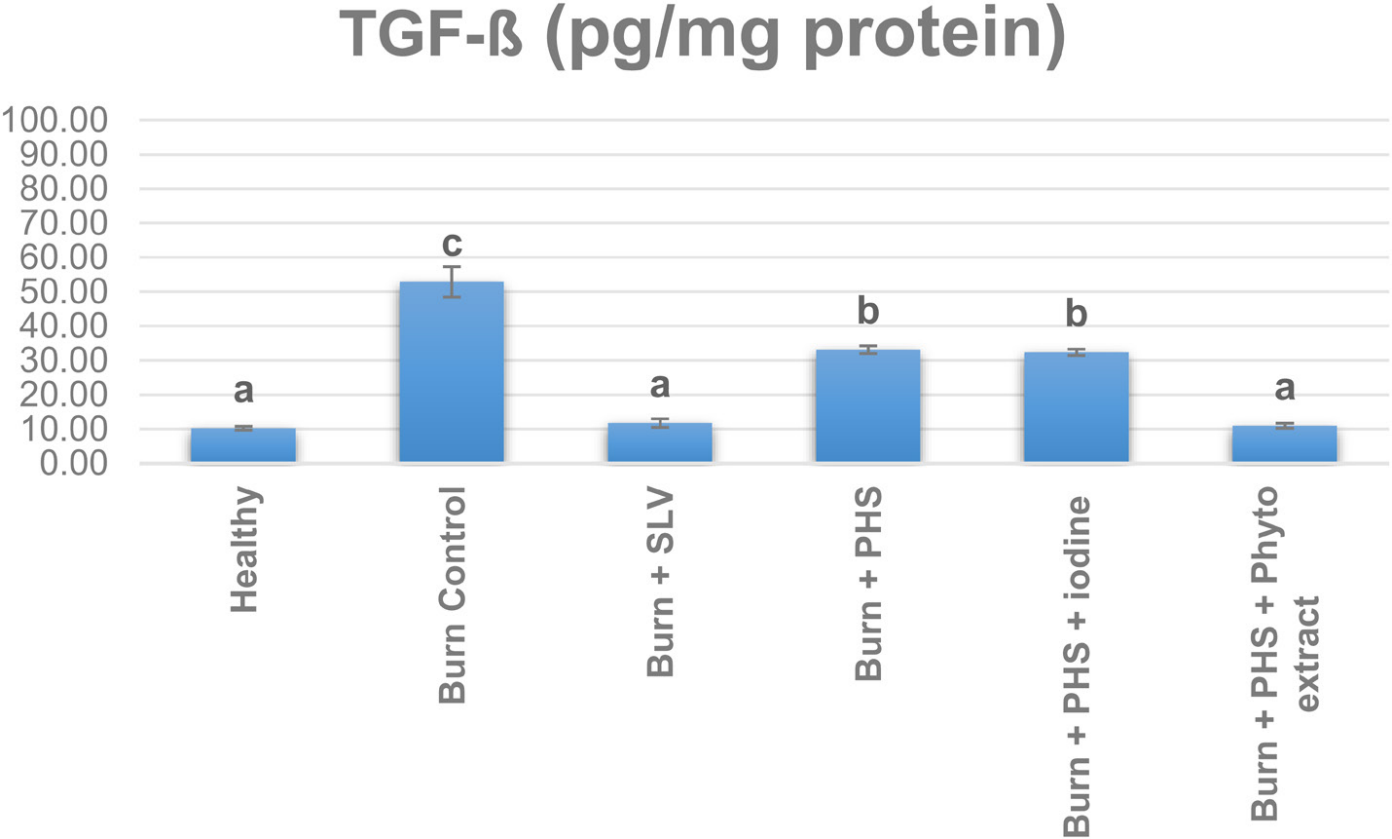
Transforming growth factor-β (TGF-β) results on skin tissue on day 21. PHS, pectin-based hydrogel system; SLV, silver sulfadiazine.

It was observed that the amount of TGF-β increased in the burn control group compared to the healthy group. This shows that the burn model is formed. When PHS and iodine groups were compared with the burn control group, TGF-β levels decreased significantly. In SLV and phyto extract groups, TGF-β levels were found to be very close to the healthy group (Figure 4).

### Vascular Endothelial Growth Factor Results

**Figure 5.**
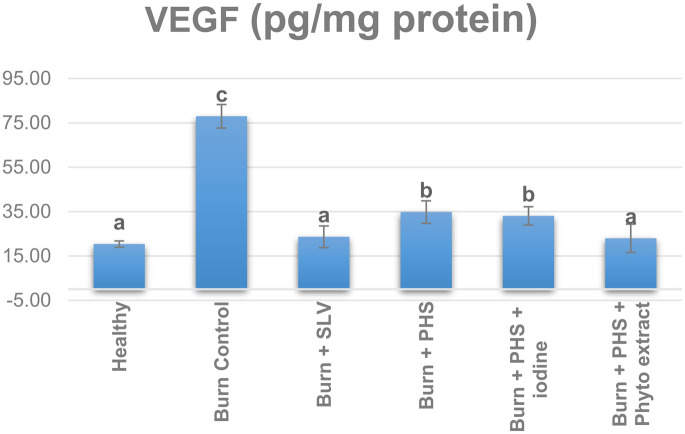
Vascular endothelial growth factor (VEGF) results on skin tissue on day 21. PHS, pectin-based hydrogel system; SLV, silver sulfadiazine.

It was observed that the amount of VEGF increased in the burn control group compared to the healthy group. This shows that the burn model is formed. When PHS and iodine groups were compared with the burn control group, VEGF levels were found to be significantly decreased, while VEGF levels in SLV and phyto extract groups were found to be very close to the healthy group (Figure 5).

### Collagen Type 1 Results

**Figure 6.**
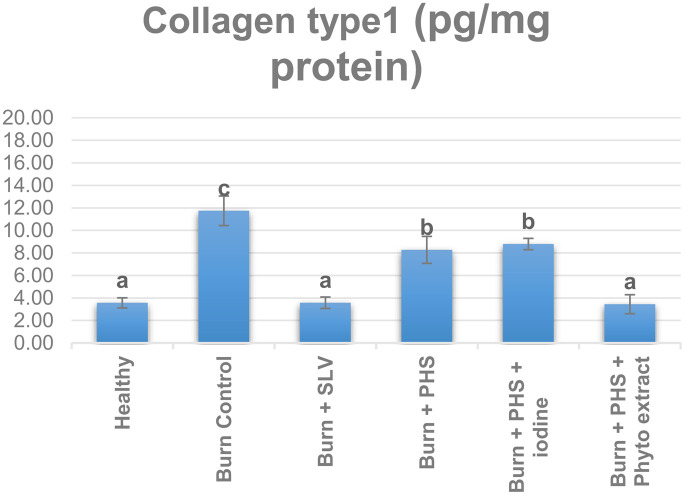
Collagen type 1 results on skin tissue on day 21. PHS, pectin-based hydrogel system; SLV, silver sulfadiazine.

It was observed that the amount of collagen increased in the burn control group compared to the healthy group. This shows that the burn model is formed. When PHS and iodine groups were compared with the burn control group, it was found that collagen levels decreased significantly; collagen levels in the SLV and phyto extract groups were very close to the healthy group (Figure 6).

### Histopathological Findings

**Figure 7.**
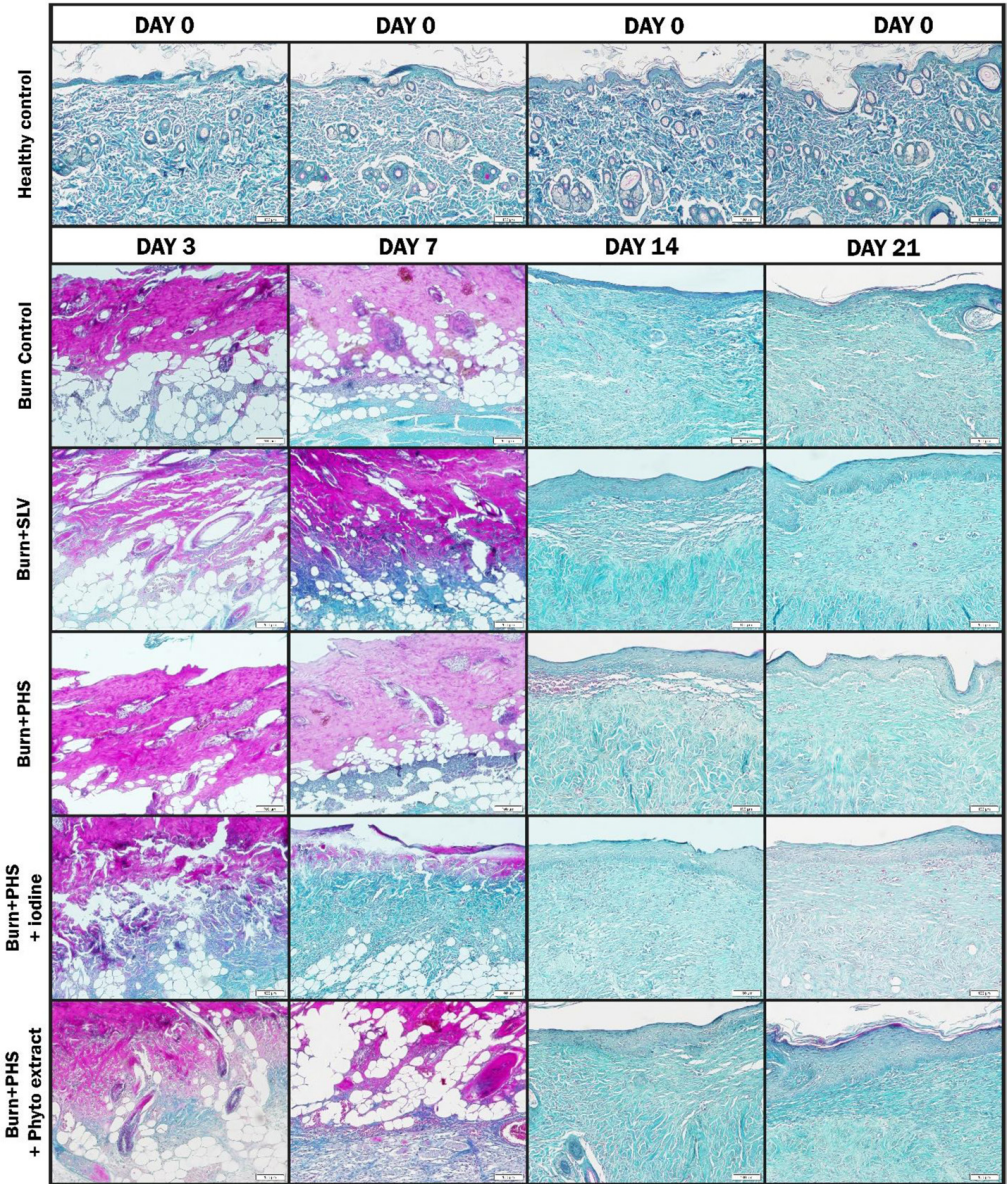
Histopatological findings on skin tissue by days. PHS, pectin-based hydrogel system; SLV, silver sulfadiazine.

When the rat skin tissue in the healthy control group was examined histopathologically, epidermis, dermis, and hypodermis layers, a thin epidermis layer consisting of 2-3 cell layers and keratin casts on the surface of this layer were observed. Irregular dense connective tissue formed by collagen and elastic fiber bundles was observed in the dermis layer. Fibroblast and fibrocyte cells belonging to connective tissue were observed in these dense fiber bundles. In addition, sebaceous glands and hair follicles were also seen in the dermis. In the hypodermis, subcutaneous fatty tissue formed by hexagonal-shaped fat cells was observed (Figure 7).

#### Burn Control Group; Day 3

A necrotic dead crust structure characterized by necrotic cells and denatured fibers was observed in the 2 main layers of the skin, dermis and epidermis. It was observed that almost all of the layers of the 2 main layers of the skin were affected by the burn. It was observed that the burn line reached almost to the border of the hypodermis. Day 7: It was observed that the burnt crust structure started to detach and separate from the dermis and hypodermis layers, and inflammatory cells were observed deep in the dermis and hypodermis. Day 14: It was observed that the dead crust layer was separated from the dermis, while granulation tissue characterized by a large number of newly developing inflammatory cells from the burn edge line towards the epidermis and dermis layer was observed to move towards the burn line. Day 21: It was observed that the number of inflammatory cells in the dermis decreased but continued to exist. It was observed that the density of collagen fibers in the dermis gradually increased and thickened the dermis, while the epidermis layer was clearly distinguished (Figure 7).

#### Burn + SLV Group; Day 3

Similar to the burn group, it was observed that almost all of the epidermis and dermis layers were affected by the burn, and the fiber structures in the dermis layer were denatured, and the cells were necrosed. Day 7: It was observed that the necrotic dermis and epidermis layers on the surface of the hypodermis were separated from each other, and there was an increase in inflammatory cells with a collagen fiber increase in the deep dermis, and granulation tissue started to develop. Day 14: It was observed that the necrotic shell structure completely disappeared, a dense granulation layer was observed in the dermis, and it was observed that the epidermis layer was formed on the new dermis rising from the deep dermis. It was noteworthy that the rate of inflammatory cells in the dermis layer was higher than the rate of collagen fibers. Day 21: It was observed that the epidermis layer was highly layered and epidermal recesses were formed from the epidermis to the dermis. While a dense collagen network was observed in the dermis, it was determined that there was no granulation layer in this area (Figure 7).

#### Burn + PHS Group; Day 3

As seen in the burn group, a characteristic necrotic crust structure was observed in this group. The burn line was similar to the other groups. Day 7: While the necrotic tissue crust was completely separated from the surface, a dermis layer dominated by dense granulation tissue was observed on the hypodermis. Again, it was observed that collagen bundles formed concentrations in the granulation tissue in this area. Day 14: It was observed that the epidermis layer was remodeled, while the granulation tissue was limited only under the epidermis. It was understood that collagen fibers in the deep and middle dermis were re-established irregularly and tightly. Day 21: In parallel with the findings of the burn group on the 21st day, prominent collagen fibers were observed in the dermis in this group. Again, it was observed that the epidermis layer developed, but dermal papillae were not yet formed. Inflammatory cell clusters were observed under the epidermis (Figure 7).

#### Burn + PHS + Iodine Group; Day 3

The second-degree burn observed in all burn groups was also clearly distinguished in this group. Burned tissue and the burn line were similar to the other burn groups. Day 7: It was observed that the necrotic tissue crust on the hypodermis line was completely separated from the surface, while dense granulation tissue developed in the hypodermis and dermis. It can be said that the dominant cell type in this area is inflammatory cells, and collagen fiber development is observed. Day 14: It was observed that the dermis and epidermis layers were remodeled, granulation tissue remained only in small amounts under the epidermis, and collagen fibers were re-established in the deep and middle dermis in a tight and irregular manner. The findings of this group were similar to the Burn + SLV group in general. Day 21: On the 21st day of treatment, it was observed that the amount of collagen in the dermis reached its highest level and no granulation tissue was observed in the dermis. The epidermis was significantly developed and thickened (Figure 7).

#### Burn + PHS + Phyto Extract Group

Day 3: In parallel with the burn groups, a similar level of burn and necrotic scab structure was observed in this group. Day 7: It was observed that the necrotic tissue crust in the dermis and epidermis was separated from the surface and granulation tissue characterized by a large number of inflammatory cells developed between the fat cells in the hypodermis and these cells dragged the fat cells towards the epidermis. It can be said that inflammatory cells are the dominant cell type in the area where granulation tissue develops. Day 14: Similar to the Burn + PHS + iodine group, it was observed that collagen fibers increased in the dermis layer. It was observed that a small amount of granulation tissue in the dermis remained only under the epidermis. It was noteworthy that the epidermis was significantly regenerated and thickened in places. Day 21: In the connective tissue remodeled in the dermis layer, irregular tight connective tissue had a typical appearance. No granulation layer was observed in this area. The appearance of the group can be said to be similar to the Burn + SLV group. The epidermis was thickened, and dermal papillae extending from the dermis to the epidermis were seen in many areas. In the uppermost layer of the epidermis, keratin fibres started to be shed in layers (Figure 7).

### Scoring of the Degree of Burn Healing

In light of the literature studies, a damage scoring system was used to better determine the skin burn healing level analysis. A compatible reference scoring system was preferred because it was intended to be similar to the literature. The relevant scoring principles are as follows.

In the evaluation of burn healing, the epithelialization of the epidermis layer and the degree of healing of the dermis layer were taken as references. This scoring is based on the deep burn wound healing process histological scoring study developed by Guo et al. (REF1: PMID ID: 33224609). During the evaluation, the epidermis and then the dermis were examined. The healing level of the epidermis was evaluated with scores ranging from 0 to 7 according to the condition of the crust on the wound surface, the formation of dermal indentations, and the degree of epithelialization. Accordingly, 0 for complete damage of the epidermis layer; 1, 2, and 3 for minimal, mild, and moderate epithelialization towards the center of the wound without separation of the scab from the edges, respectively; 4 and 5 for complete separation of the scab and moderate and severe epithelialization towards the center of the wound; 6 for complete coverage of the wound surface with a newly developing epithelial layer and 7 for the appearance of dermal papillae.

At the level of burn healing observed in the dermis layer, the rates of inflammatory cells, fat cells, fibroblast cells, hair follicles, and collagen deposits were evaluated by scoring between 0 and 7. In this scoring, 0 for the presence of predominantly fat cells and few inflammatory cells in the dermis; 1 for a decrease in the proportion of fat cells and a moderate presence of inflammatory cells; 2 for the presence of a few fat cells and a significantly higher proportion of inflammatory cells; 3 for a further increase in the proportion of inflammatory cells; 4 for a combination of collagen fibers and an increased proportion of inflammatory cells in the dermis; 5 for the absence of fat cells, a moderate level of inflammatory cells, and an increase in the number of fibroblast cells and collagen deposits; 6 for a small number of inflammatory cells and a large number of fibroblast cells in the dermis; and finally, 7 for the observation of immature hair follicles and a high level of collagen deposition (Table 2).

## Discussion

This study investigates the efficacy of PHS in the treatment of burn wounds and the effect of adding iodine and plant extracts to these systems. In the experiment, 60 Wistar albino rats were used, and different treatment groups were analysed. These included healthy control, burn control, treatment with SLV, treatment with PHS, a combination of PHS and iodine, and a combination of PHS and plant extracts. Plants have a wide potential in the management and treatment of wounds and burns with their antioxidant, anti-inflammatory, and antimicrobial activities.^20^ Due to the waning of pharmacological effects of some plant extracts, we investigated a new pharmaceutical transdermal system prepared from pectin and plant extracts.

Pectin-based hydrogel systems (PHS) is a hydrogel system prepared with pectin-gelatin complexes and produced by a new patented method. An iodine-containing solution was added to PHS to incorporate antibacterial properties. Similarly, plant extracts were also combined with PHS. In the experiment, the treatment was applied for 21 days, and the healing process of the wounds was observed macroscopically. The efficacy of the treatments was evaluated by macroscopic, biochemical, and histopathological analyses. In biochemical analyses, TNF-α, TGF-β, VEGF, and collagen levels were examined. In histopathological examinations, the healing process of skin tissue was evaluated in detail.

Wound healing is a complex process that occurs to restore the structure in the injured tissue and to return the damaged tissue to its normal state as soon as possible.^21^ Wound healing has 3 phases: inflammation, proliferation, and remodeling of the extracellular matrix. Angiogenesis, collagen deposition, epithelialization, and wound contraction all occur during the proliferative phase.^22^ The healing process aims to prevent pathogen infiltration, confirm the integrity of injured tissue, and restore the physiological function of the skin.^22^

Histopathological analysis is the most important parameter in the follow-up of burn wounds. Histopathological analyses are also one of the important indicators to show the healing stages of the skin layer and to evaluate the treatment follow-up.^23^ According to our histopathological evaluation, it was observed that Burn + SLV, Burn + PHS, Burn + PHS + iodine, and Burn + PHS + phyto extract treatment groups showed regenerative effects in the dermis and epidermis layers on the 14th day. Especially in the SLV and phyto extract treatment groups, significant improvement was observed in the development of granulation tissue and reformation of collagen fibers. Previous studies have shown that maintaining skin moisture accelerates wound healing.^24^ Similarly, our findings suggest that PHS contributes to wound healing by maintaining skin moisturization.

In this study, we also analyzed growth factors known to play roles in proliferation, angiogenesis, granulation tissue formation, and wound healing process.^23,25,26^ Vascular endothelial growth factor (VEGF) is involved in angiogenesis, which promotes endothelial cell migration and proliferation and accelerates wound healing, which is important in the skin regeneration process in the early stages of wound healing.^27^ Transforming growth factor-β (TGF-β1) plays a key role in maintaining skin homeostasis.^28^ A previous study showed that VEGF-A and TGF-β1 levels in burned skin increased significantly from the 7th day after burn injury and then continued to decrease and approached normal levels after 28 days of treatment.^29^ Similarly, in our study, we showed that VEGF and TGF-β1 levels increased in the burn group on day 21 and decreased in the treatment groups in parallel with healing.

Angiogenesis is initially regulated by various factors such as VEGF and TGF-β. Another protein that strongly stimulates this process is type I collagen. Collagen is the most common protein in the human body and contributes to the mechanical strength and elasticity of tissues. It can mediate many pro-regenerative physiological interactions during the wound healing process, from angiogenesis to re-epithelialization.^30^ During the healing of burn wounds, it promotes scar formation by abnormal and excessive collagen deposition and increased thickness of the epidermis and dermis in the defect area.^31^

In a previous study, Tran et al.^32^ showed that Col1 accumulation, which increased in burns, decreased with treatment and this decreased scar formation. In our study, similar to previous studies, Col1 levels, which were increased in the burn control group, decreased in all treatment groups.

Studies have suggested that TNF-α is an important indicator of poor prognosis after thermal injury.^33^ Tumor necrosis factor-alpha (TNF-α) is mainly produced by activated macrophages and regulates the production of some other cytokines, increases endothelial adhesiveness for leukocytes, stimulates neutrophils and monocytes, and promotes their adhesion, phagocytosis, and degranulation.^34,35^ In a study by Deveci et al.^34^ it was shown that TNF-α, which was increased in burn control groups, decreased with treatment. In our study, it was observed that TNF-α level was high in the burn control group and decreased in the treatment groups.

In conclusion, this study demonstrates the potential of new treatment methods, such as the combination of PHS with iodine and phyto extracts, in the burn healing process and provides a basis for future research. The results of the study show the efficacy of PHS, a new treatment method, and suggest that herbal extracts, as well as traditional treatments, can contribute positively to the burn healing process. This study may contribute to the development of more effective and innovative approaches in the field of burn treatment in clinical practice.

The lack of molecular analyses may be an important limiting factor of this study. This study may lead to clinical studies with pectins obtained from different plants and fruit species as a natural treatment method.

## Data Availability Statement

The data that support the findings of this study are available on request from the corresponding author.

## Figures and Tables

**Table 1.** *In-vivo* Experimental Groups

Groups	Number of Rats
Healthy control	10
Burn Control	10
Burn + SLV	10
Burn + PHS	10
Burn + PHS + iodine	10
Burn + PHS + phyto extract	10

PHS, pectin-based hydrogel system; SLV, silver sulfadiazine.

**Table 2.** Histopathological Scoring between Groups by Days

Groups	Epidermis Healing				Dermis Healing			
	3rd day	7th day	14th day	21st day	3rd day	7th day	14th day	21st day
Healthy control	7	7	7	7	7	7	7	7
Burn control	0	1	4	6	0	1	5	6
Burn + SLV	0	2	5	7	0	2	6	7
Burn + PHS	0	1	4	6	0	2	5	6
Burn + PHS + iodine	0	2	6	7	0	3	6	7
Burn + PHS + phyto extract	0	2	6	7	0	3	6	7

PHS, pectin based hydrogel system; SLV, silver sulfadiazine.
